# Deontological or Utilitarian? An Eternal Ethical Dilemma in *Outbreak*

**DOI:** 10.3390/ijerph18168565

**Published:** 2021-08-13

**Authors:** Po-En Tseng, Ya-Huei Wang

**Affiliations:** 1 Graduate Institute of International Human Resource Development, National Taiwan Normal University, Taipei 106, Taiwan; thpss28537584@gmail.com; 2 Department of Applied Foreign Languages, Chung Shan Medical University, Taichung 402, Taiwan; 3 Department of Medical Education, Chung Shan Medical University Hospital, Taichung 402, Taiwan

**Keywords:** medical ethics, deontological ethics, utilitarian ethics, ethical dilemma, public health

## Abstract

Both deontological ethics and utilitarian ethics are important theories that affect decision making in medical and health care. However, it has been challenging to reach a balance between these two ethical theories. When there is a conflict between these two ethical principles in the medical context, the conflict must be addressed in order to reach an appropriate solution for patients and others involved. To demonstrate decisions made in terms of deontological ethics and utilitarian ethics, the study will use the film *Outbreak* as example to further understand these two ethics in relation to epidemiology and public health. The paper will also analyze film scenarios to examine how deontological ethics and utilitarian ethics are involved and strike a balance with different pearspectives to reach an appropriate public health solution. To reach more just solutions, it is essential to determine how to make wise decisions by balancing deontological ethics and utilitarian ethics. However, the decision-making process is complicated because any solution must consider not only medical ethics but also political, environmental, and military issues. In order to reach an appropriate public health decision, those involved should be inclined toward empathy and contemplate things from different ethical perspectives to deal with ethical/moral dilemmas and create greater beneficence and justice for patients and humanity at large.

## 1. Medical Ethics and Ethical Principles

Medical ethics is a crucial factor in making decisions regarding any moral or ethical dilemma or conflict, as well as the resulting consequences that medical care professionals may face [1]. Ethics refer to not only the study and practice of moral choices involved in moral values, but also the judgments behind those moral choices that justify moral decisions [2]. Before the 1970s, there was no bridge connecting philosophical ethics with medical ethics; however, since the 1970s, scholars have considered medical ethics in connection with public health and nursing [3].

Although there were no formal medical ethics principles or theories before the 1970s, doctors and medical care professionals still had moral values on which they relied when treating patients. Medical ethics stem from the tradition of Hippocratic Oath [3], also known as the physician’s oath, to deal with the ethical dilemmas or conflicts that healthcare professionals might encounter in a healthcare context [4]. During the Second World War, the inappropriate treatment and even mistreatment of human subjects in medical experiments facilitated *The Belmont Report* [5] and the start of principled ethics to confirm the ethical principles in biomedical and human behavioral research. To ensure biomedical safety and patient rights, The Belmont Report [5] presented three core principles: autonomy, beneficence, and justice. Autonomy refers to respect for persons from two perspectives. First, each individual should be apprised before any treatment or experiment; second, it is necessary to protect any person whose autonomy is diminished. Beneficence is related to respecting to every person’s decisions, protecting them from harm, and ensuring their well-being. Justice refers to the idea that resources, efforts, contributions, and merits should be equally allocated to people no matter their race, religion, gender, etc.

## 2. Ethical Principles and Ethical Dilemmas: Deontological Ethics and Utilitarian Ethics

Beauchamp and Childress [6] proposed four ethical principles to ensure patients’ rights, and these are the basis for ethical principles in medicine. The principles are autonomy, beneficence, non-maleficence, and justice. According to Beauchamp and Childress [6], the autonomy principle refers to the obligation to respect people who are capable of making decisions and taking actions based on their personal views and values. The beneficence principle refers to the obligation to consider people’s best interests and to act so as to increase their welfare. The non-maleficence principle refers to the obligation to not intentionally harm people. The justice principle refers to the obligation to treat each person fairly and equally, whether in benefits or in risks. No principle takes priority over the other principles. That is, the four principles share equal weight, and no one is more significant that the other in terms of moral decision making [7].

The four principled ethics have served as the codes for healthcare professionals to prevent disease and facilitate medical care. Although the principled ethics have detailed regulations, in real clinical or medical practice, doctors or medical professionals often face ethical dilemmas regarding how to incorporate the four ethical or moral principles to derive a comprehensive plan for treatment [8]. When making clinical judgments, medical professionals should focus not only on patients but also on potential patients who may be in danger or be infected. Hence, these four moral principles create difficulties in arriving at a coherent moral judgment/resolution for medical care professionals. Therefore, to make wise clinical decisions, medical care professionals must simplify their moral judgment processes to reach a coherent justification for the sake of medical practice and public health.

Medical ethics deals with conflicts, dilemmas, and choices regarding obligations, morality, and public interest. Deontological ethics and utilitarian ethics are both ethical theories and dominate decision making in medical care and health care [4]. Deontological ethics are inclined to be patient-centered; hence, consequences are not used to justify means. However, utilitarian ethics, which are inclined to be more society-centered, value care for the greatest welfare for the greatest number of human beings; hence, outcomes determine means [1]. In clinical practice, doctors and other medical care professionals may rely on these two strands of ethical theory to make medical or clinical decisions. In recent years, scholars have observed the conflict between deontological ethics and utilitarian ethics, and it has caused some frustration and discontent. Moreover, a medical decision based on deontological or utilitarian ethics may cause conflicts in medical ethics and conflicts between doctors and patients. Although deontological ethics and utilitarian ethics differ, both have their strengths and weaknesses in medical practice. It is not easy for doctors and other medical care professionals to find a balance between these two ethical approaches.

The conflict between deontological ethics and utilitarian ethics is more obvious when an epidemic or pandemic breaks out that endangers public health. Theoretically, when a conflict of two or more ethical theories occurs in a medical issue, healthcare professionals and stakeholders should reflect on the issue in order to reach an appropriate decision. However, in practice, one ethical theory may override the other, causing a controversy in medical practice. Healthcare professionals must therefore critically reflect on these ethical issues to develop decision-making abilities that best help patients, patient families, physicians, and other healthcare professionals [4].

As there is no definite solution to ethical issues, in order to further understand these two ethics in epidemiology and public health, the study will use the film *Outbreak* as example to demonstrate the differences between deontological ethics and utilitarian ethics and examine the dilemma between these two ethics in social, economic, and business spheres. The paper will also analyze film scenarios to examine how deontological ethics and utilitarian ethics are involved, and further reflect upon the present scenario of the COVID-19 outbreak into a global pandemic to contemplate how medical professionals or decision makers can manage to strike a balance with different perspectives to reach an appropriate public health solution.

## 3. The Deadly Epidemic in *Outbreak*

The film *Outbreak* was directed by Wolfgang Peterson [9], and was based on Richard Preston’s 1994 nonfiction book, *The Hot Zone* [10]. *Outbreak* [9,11] describes the unknown Motaba virus, discovered in a jungle in Africa in 1967, during the Kisangani Mutinies. People infected with the Motaba virus would have deadly fevers and die quickly and painfully. In *Outbreak*, Dr. Raswani (Malick Bowens), a black African army physician, has tried to make his patient, an Australian mercenary infected with Motaba virus, comfortable. However, Raswani and his nurse are helpless and can only watch the patient. Suddenly, the mercenary patient screams sharply and begins convulsing, his eyes rolling back; then, he dies. An American captain promises Raswani that he will send a plane with supplies, doctors, and nurses. However, when the captain sees the new Motaba virus spreading even faster and almost wiping out a village, instead of sending help, the captain sends an airplane with a firebomb to incinerate the village.

Nobody knows where the Motaba virus comes from. The audience learns that there is an army conspiracy involving the use of the lethal Motaba virus as a bioweapon, a scheme developed by two U.S. Army officers, Brigadier General Billy Ford (Morgan Freeman) and Major General Donald “Donnie” McClintock (Donald Sutherland). To keep the bioweapon scheme a secret, after taking blood samples from a dying American mercenary, Ford and McClintock let the American captain firebomb the camp in the African jungle where soldiers had been infected and not infected in order to eliminate the Motaba virus infection.

Major General McClintock and Brigadier General Ford assume that the firebombing wiped out the outbreak of the Motaba virus. However, somehow the deadly Motaba virus has survived, spreading from one carrier to another. Twenty-eight years later, in 1995, the deadly Motaba virus resurfaces in Zaire. Smuggler James “Jimbo” Scott (Patrick Dempsey), working for a bio-tech animal laboratory, illegally imports an African monkey carrying the deadly virus, Betsy, to the United States. Soon, the Motaba virus spreads to Boston and then California. Brigadier General Ford assigns Colonel Sam Daniels (Dustin Hoffman), a virologist in the United States Army Medical Research Institute for Infectious Diseases (USAMRIID), to investigate the case. In the draft script of *Outbreak*, written by Dworet and Pool (1993), Colonel Sam Daniels is named Gillespie. In the film of *Outbreak*, directed by Peterson (1995), the character is named Colonel Sam Daniels. For coherence, this study uses the name Colonel Sam Daniels throughout the paper.

Army doctors, including Colonel Daniels, Lieutenant Colonel Casey Schuler (Kevin Spacey), and Major Salt (Cuba Gooding Jr.), begin investigating a cure for the Motaba virus. The virus has now mutated into an airborne virus and begun spreading through a little town in California: Cedar Creek. A number of Cedar Creek residents are exposed to Motaba at a theater and become infected. To stop the virus from spreading, Daniels requests a quarantine in Cedar Creek, while Major General McClintock, Brigadier General Ford’s boss, with the approval of the U.S. president, orders Ford to bomb Cedar Creek to stop the outbreak of Motaba, as they did twenty-eight years earlier in the African jungle. In time, the host monkey Betsy is captured, and Ford delays the bombing to allow Major Salt time to create an antiserum to save the Cedar Creek residents. With the outbreak of Motaba virus in the little town of California, there is an ethical tension and dilemma between utilitarianism and deontology.

## 4. Utilitarian Ethics

The founder of modern utilitarian ethics, Jeremy Bentham, introduced in *An Introduction to the Principles of Morals and Legislation* [12,13] the principle of utility for the evaluation of appropriate actions. The rightness or wrongness of a selected action is decided according to whether the action would maximize a positive outcome, that is, whether the action would bring less pain and more pleasure to the most people. Bentham [12,13] quantifies the amount of pain and pleasure created from actions in a moral utilitarian calculus that examines the rightness or wrongness of the selected actions in terms of seven factors: intensity, duration, certainty, propinquity or remoteness, fecundity, purity, and extent [14].

Utilitarian ethics is a version of consequentialist ethical theories. Although there are different varieties of utilitarian ethical principles, the basic idea of these principles is based on Bentham’s theory: maximize utility and prioritize public happiness. Bentham [12,13] believed that the greatest happiness of most people is the criterion that should be used to judge the rightness or wrongness of actions. For instance, the most correct decision is to sacrifice a few people to achieve public happiness, even if we sacrifice them in a merciless or brutal way. Utilitarian ethics has been applied not only to social welfare economics but also to the most recent financial crisis [15]. However, Bentham’s quantitative utilitarianism has been criticized. People have begun to question about the concept of hedonic calculus because the so-called “maximum happiness” and “minimum pain” can be subjective and hence have difficulty in calculating the greatest happiness for the greatest number [16].

In *Outbreak*, Brigadier General Ford and Major General McClintock follow utilitarian principles. While seeing that the Motaba virus is out of control in the African jungle, they decide to incinerate the village. They believe that there is no essential rightness or wrongness of bombing; the so-called rightness or wrongness of bombing depends on the whether the bombing will have a positive consequence, that is, whether the bombing will wipe out the Motaba virus infection or not. Hence, as in Bentham’s [12,13] moral utilitarian calculus, in order to bring less pain to the most people, McClintock and Ford, in the helicopter, look out “the window at the campsite”:

Major General McClintock: It’s viral. There’s no way to stop it. It could spread all over the world. If you’ll excuse my bluntness, sir, you cannot go halfway on this one.

Brigadier General Ford: Do not ever—ever—ever—tell me what I have to do [11] (p. 3).

After that, while the soldiers celebrate that the plane has come with supplies, McClintock, in the copilot’s seat, puts his “hand on the green bomb release lever,” saying “It’s either them or us” [11] (pp. 4–5). Then, the bomb explodes, causing screams of agony and the deaths of Raswani and the soldiers.

Utilitarian ethics originated with the idea of making good use of time and resources in medical care, without taking public benefit into consideration. However, utilitarian ethics evolved to mean a decision based on the maximum benefit for the greatest number of human beings [1]. However, when utilitarian ethics uses the maximum benefit for the most people as its primary consideration, some individuals or groups may be harmed.

In *Outbreak*, the Motaba virus cannot be cured unless the host is found. People infected suffer from a deadly hemorrhagic fever, and become pale and start to cough; within a few hours, a patient’s eyes roll back and they convulse and die in agony. When there is no hope of finding the host, in order to stop the virus from spreading, the U.S. government orders the military to take over from medical professionals in order to stop the spread of the virus. The U.S. Army quarantines Cedar Creek to repress the Motaba virus outbreak, taking actions to prevent Cedar Creek residents from breaking quarantine, saying “Go home and stay there or you will be placed under arrest.” [11] (p. 77). From a utilitarian perspective, in order to stop the infection, the soldiers do not care about the residents’ panic, trying to shoot a resident who wants to flee at all costs. To fulfill their objective of quarantining the residents to prevent infection, the soldiers do not care about the residents’ terror.

Major General McClintock orders a quarantine to stop the virus from spreading. This order does not respect the rights of patients. Moreover, in consideration of how to enact most overall good, the government intends to sacrifice a few people, with no intention to distribute medical resources to those in need. Furthermore, the government intends to kill the residents, even residents who are not necessarily infected. This decision not only violates the non-maleficence principle but also the justice principle. In *Outbreak*, the residents do not have the antidote; moreover, without enough medical aid, the patients wait to die in hospital without care. Though the quarantine causes panic and horror for residents of Cedar Creek, brutally and mercilessly sacrificing Cedar Creek residents would prevent others from being infected and thus achieve the greatest happiness to the public. Moreover, to root out the infection of the Motaba virus, with the approval of the U.S. president, Major General McClintock orders General Ford to bomb Cedar Creek. Thus, the actions (quarantine and bombing) that lead to maximum social welfare would justify the decisions and actions [15]. From a utilitarian perspective, medical resources are finite; hence, there is a need to appropriately distribute them to reach the maximum health care benefit for the greatest number of human beings [1].

## 5. Conspiracy under the Mask of Utilitarianism in *Outbreak*: Biowarfare and Utilitarian Ethics

The army has an antidote for the Motaba virus. However, to keep using the Motaba virus as a bioweapon, McClintock and Ford do not want to use the antidote to save the soldiers infected in the camp. This decision causes the disease to spread. Not caring about the greatest benefit of most people, McClintock and Ford let the infection break out: “It’s viral. There’s no way to stop it. It could spread all over the world” [11] (p. 4). Only when the infection spreads out of control can McClintock and Ford begin to think about the greatest benefit of most people. They use the bomb to clean the infection; McClintock “squeezes the green lever. The bomb cannisters under the wing open up, releasing a thick cloud of yellow cyanide gas”, causing screams “of agony rip out as Raswani and the soldiers convulse and die” and “the cries of animals caught in the fire” [11] (pp. 4–5). Using the bomb is an act of utilitarianism, bringing the greatest benefit and least pain to human beings.

However, there is a biowarfare conspiracy under the mask of utilitarianism. While examining the charred wreckage of the van, Brigadier General Ford and Colonel Daniels are appalled. Daniels censures Ford’s immoral behavior of using the Motaba virus for biowarfare and a catastrophe for human beings. Daniels says: “You had the antiserum to the original African strain, you could’ve made it available right away. The virus would’ve never had a chance to mutate. You could have stopped this whole epidemic before it got started. Why?” [11] (p. 100).

Thinking that humans have the power to control nature, McClintock and Ford perform biowarfare research to create the maximum welfare for their own country, without thinking that the research will sacrifice innocent people. Nature responds by devouring human beings, taking over control from the research. However, though with “sadness and remorse”, and in order to hide the biowarfare research, Ford gives an order to kill Iwabi’s people in Africa. While Daniels accuses Ford, asking whether he “ordered the attack on Iwabi’s people in Africa”, Ford, neither confirming nor denying the accusation, tries to justify the reasonableness of attacking Iwabi’s people. There is an intense debate between Daniels and Ford:

Colonel Daniels: You ordered the attack on Iwabi’s people in Africa?

Brigadler General Ford: We had to ensure that we had the only live samples of the virus.

Colonel Daniels: Biowarfare research is illegal. We signed a treaty...

Brigadler General Ford: …What do you know about the real world, Gillespie [Daniels]? Even the air you breathe is artificial. Why do you think Saddam Hussein did not kill our boys with his anthrax and his botulism and all the other crap he created in his labs? ’Cause he knew we had something worse—

Colonel Daniels: One mad man.

Brigadler General Ford: Every fucking dictator of the future’s going to use biological weapons that you can manufacture cheap in a test tube with technology you can buy at the newsrack. Deterrence works [11] (pp. 100–101).

After hearing Ford’s conspiracy under the mask of utilitarian philosophy regarding using the Mutaba virus as weapon to attain a military advantage, Daniels is horrified. Only at this moment does Daniels realize that he is involved in a biowarfare conspiracy and is a victim of biowarfare research. To keep the virus conspiracy as a secret, under the mask of utilitarianism, Ford presumptuously says: “We gambled. We lost. We cannot afford to lose again...”, to justify the rightness of his bombing behavior” [11] (p. 101). Moreover, to cover up the Mutaba virus biowarfare program, McClintock arrests Daniels as a carrier of the Mutaba virus, deterring Daniels from finding a cure for Mutaba virus.

## 6. Utilitarianism to the Extreme: Brutality and Inhumanity

To secure the biowarfare program, under the mask of the utilitarian principle, the characters use a bomb to eradicate the infection. Wiping out the camp seems to cause less pain and greater welfare for the public; most of all, the action will forever keep the bioweapon conspiracy a secret.

Colonel Daniels: What if there were man who could stopped it, but did not? How would history judge such men?

General Major McClintock: Truman dropped the bomb on the Japanese. Saved hundreds of thousands of Americans lives. Now revisionist say he dropped the bomb to scare the Russians

Colonel Daniel: Those men were at war. We are not.

General Major McClintock: We are at war. Everybody is at war.

Colonel Daniel: These people are Americans.

General Major McClintock: 2600 dead or dying Americans. If the virus gets out of there, 260 million Americans will be dead or dying. Those people are casualties of war [9] (pp. 72:38–73:33).

In this epidemic, when utilitarianism goes to the extreme, people may use biotechnology or atomic bombs under the mask of utilitarian philosophy, not to do most good overall but to secure the greatest benefits to themselves or their countries, without caring for innocent victims. In other words, when utilitarians go to extremes, those involved in a biowarfare program to attain a military advantage would not care about human suffering but instead use brutality and inhumanity, sacrificing innocent people. From a utilitarian perspective, the use of an atomic bomb to eradicate the Motaba virus could save the most time and resources. However, the costs of sacrificing innocent lives and ignoring human suffering are considerable.

## 7. Deontological Ethics

The philosopher Immanuel Kant [17,18,19] introduced the concept of deontological ethics; hence, deontological ethics is also called Kantian deontology. Being a devout Christian, Kant grounded his duty-based ethical principles in terms of universal moral obligations. Moreover, thinking that each human being has an inherent value, Kant thinks that the autonomy, dignity, and respect concerning each individual should be emphasized. Ross [20] modified Kant’s deontology, allowing a plurality of duty-based ethical principles, such as doing no harm, promise keeping, etc.

In contrast to utilitarian principles, deontology principles refer to the ethics of duty, in which no harm is allowed, even if it may lead to positive consequences [1]. Hence, decisions made based on deontological ethics may be appropriate for an individual even though those decisions may not lead to good outcomes for society [1].

Unlike utilitarianism, in which the justification for an action is decided by whether the action would bring the greatest happiness/welfare for the most people or society, in deontology, whether an action is moral is evaluated by the nature of the action, not its consequences. To win the war (consequence), Major General McClintock and Brigadier General Ford developed the Motaba virus and kept it as a biological weapon (action). However, the action of developing the Motaba virus and keeping the virus as a biological weapon is inherently wrong and causes numerous deaths. Hence, the development of the Motaba virus violates both utilitarian ethics and deontological ethics.

Deontological ethics emphasizes the value of being a human being, underlining the principles of respect for autonomy, beneficence, non-maleficence, and justice [21]. Therefore, deontological ethics can help medical care professionals further understand the four principles, regarding respect for autonomy, non-maleficence, and justice as the principles of humanity values and beneficence as a principle of maximizing human happiness and relieving suffering [21].

People have a duty to act in the right manner, even they risk harming themselves. According to Kant [17,18,19], doing what is right is not grounded in the consequences of actions but in the good intention in taking said actions. Hence, duty and obligation would be the basis for deontological ethics in making a moral decision. In other words, people have to abide by their moral duty or obligation rather than by the consequences of their actions. However, in order to act in the right manner, healthcare professionals sometimes risk becoming infected. As in *Outbreak*, while Colonel Daniels mentions filovirus infection in a human being, he warns Major Salt:

You’ll be holding a needle and it’ll slip. Or your glove will have a crack in it and you will not notice. You work with filoviruses, it’s like working with plutonium. A single drop of blood can hold six billion... That’s more filoviruses than there are people in the world. You get a single one of those inside you, you’re infected. Say you’re lucky and it’s one of the few filoviruses we have antiserum for. Then, we can treat you and you’ll probably live. However, say you’re unlucky. Additionally, you get one of those filoviruses we do not have an antiserum for, which is most of them. There’s no medicine, no cure, nothing we can do to help you. Your body gets so hot, your liver, your kidney, all your vital organs melt, and your skin turns into tapioca pudding.

[11] (p. 19)

Daniels, Schuler, Salt, and Dr. Roberta “Robby” Keough (also Colonel Daniels’ ex-wife; played by Rene Russo) investigate and find a cure for the deadly virus. They understand the horror of the Motaba virus and that they may be infected. For instance, a lab technician in Cedar Creek is infected when he inadvertently breaks a vial of Rudy’s blood. Robby inadvertently pricks herself with an infected needle. Knowing that the Motaba virus has mutated into an airborne virus and has begun spreading throughout Cedar Creek, and realizing that there is no hope of quickly finding the host, all Daniels can do is to set up a quarantine in Cedar Creek to stop the virus from spreading. The action of setting up a quarantine in Cedar Creek is right, because the intention is to stop the outbreak of Motaba virus. However, though Major General McClintock supports the quarantine but imposes martial law, the nature of his action is wrong because he plans to bomb all Cedar Creek residents. Thus, being a deontologist, prioritizing the “right action” over a “good consequence”, Daniels insists on doing the right thing. Here, the right thing is setting up a quarantine in Cedar Creek to stop the virus from spreading. Moreover, Daniels tries to stop McClintock from firebombing the Cedar Creek residents because firebombing is wrong and against deontological principles, in that it is wrong to kill innocent people.

Additionally, after all, without finding the host, it is impossible to root out the infection; instead, the virus has a chance to mutate to airborne transmission.

Colonel Daniel: Therefore, you’re going to wipe out this entire town—kill everyone in it?

Brigadier General Ford: The virus is going to reach the river.

Colonel Daniels: Take the people who are well and get them out!

Brigadier General Ford: If even one were sick, no matter where we were, the disease would start again.

Colonel Daniels: Your only hope is to look for the host.

Brigadier General Ford: I did—for thirty years.

Colonel Daniels: Let me.

Brigadier General Ford: You might be contaminated.

Colonel Daniels: I know I’m not.

Brigadier General Ford: We have facilities to hold a few people in quarantine. Get out now. Take your team.

Colonel Daniels: Izzy is sick, Robby infected.

Brigadier General Ford: I’m sorry.

Colonel Daniels: Wipe out the town, wipe out Motaba. Additionally, you’re still the only one in the world who’s got the virus. No matter what, your program’s secure.

Brigadier General Ford: That’s not a consideration.

Colonel Daniels: Bullshit! [11] (pp. 100–102).

The only way to root out the infection is to find the host in order to develop the antiserum for the Motaba virus. As Daniel, a deontologist, says, “If we can find the host, we can obtain an effective antibody against the virus, and reproduce it. We can present them with a credible alternative...” [11] (p. 114).

Moreover, to protect the citizens in Cedar Creek from being infected, against Brigadier General Ford’s orders, Daniels flies to Cedar Creek, putting himself in danger to help to find the cure for the Motaba virus. Moreover, Daniels tries to intervene in McClintock’s plan to bomb the Cedar Creek residents. All Daniels’s actions stick to the principles of autonomy, non-maleficence, and justice, which refer to humanity values, and the principle of beneficence, which refers to maximizing humans’ happiness and minimizing their suffering [21].

Deontological ethics initiated from humans’ common moral principles or obligations that can be rationally deduced; hence, people can intuitively identify scenarios that are immoral. For instance, it is immoral to kill infected people in order to save people who are not infected. Moreover, as for those infected already, deontologically, medical care professionals should do their best to help them live with dignity, at least not cause harm, and treat them with respect and empathy while performing their moral and clinical duties. However, there may be some disadvantages to deontological ethics in a catastrophic situation. For example, if there were a rapid and predictable spread of infectious disease that would definitely kill thousands or millions of human beings, is it justified to sacrifice few infected and incurable people in order to save millions of those not infected? If the answer is “yes”, such behavior goes against the code of medical commitment. Based on deontological ethics, medical professionals should be committed to providing medical care to protect humans from any disease or injury, even if an epidemic is out of control. To secure each individual’s well-being, medical professionals should protect each individual from being hurt. Moreover, if they must intervene to control an epidemic, medical professionals should try to minimize the harm that may occur from the treatments or experiments. Additionally, Communicable Disease Control must stipulate prevention strategies or acts to protect people from being infected. However, these interventions may somehow deprive people of their freedoms, such as requests by border control, social distancing, and wearing masks in the community, in order to protect the public and slow down the spread of the pandemic in the community [22]. Moreover, those infected or in contact with confirmed cases will be quarantined or isolated. Though these measures may pay less attention to people’s autonomy, the measures respect each human being with an inherent value inside and thus aim to bring no harm to each individual in order to promote the best interests of human beings. As each individual is respected, they would never be sacrificed under the deontological ethical principle. In contrast, in *Outbreak*, a whole village is sacrificed in order to stop the spread of the Motaba virus in terms of utilitarian ethical principles.

## 8. Deontological or Utilitarian? An Eternal Ethical Dilemma

In *Outbreak*, many scenarios present a conflict between deontological and utilitarian ethics. The following scenario gives such an example.

Colonel Daniels: Why keep me out of there?

Brigadier General Ford: It’s a civilian matter. CDC is on it. Let them do their job. Besides, we do not have a charge.

Colonel Daniels: F**k the charge. People are dying. It’s about you being a doctor. It’s about that sacred oath we took! Remember? [9] (pp. 44:06–44:21).

Brigadier General Ford thinks order is more important than people’s lives. However, this act is against the doctor’s oath and basic medical ethics. According to the Hippocratic Oath [23], doctors should do their best to treat the ill, to preserve their privacy, and keep them from harm and injustice. Hence, Colonel Daniels, based on deontological ethical principle, condemns Ford, saying that he not only forgets a doctor’s duties but also the sacred oath that every doctor should take. The goal of medicine is to maintain health and, if possible, prevent disease and injury. As for those ill, medicine aims to treat their disease and at least relieve their suffering [23,24]. As medicine deals with duties, obligations, and moreover, moral conflicts or dilemmas, ethics play a crucial role in guiding good medical practice in terms of four fundamental principles: autonomy, beneficence, nonmaleficence, and justice. Hence, with patients’ best interest as the primary goal, in medical practice, doctors should not violate the four fundamental principles.

However, in the spread of pandemic disease, what if the development of medical ethics and epidemiology cannot prevent viruses from evolving and mutating? Eventually, virus evolution may surpass the speed of human research and the creation of vaccines. When that day comes, the deontological principle may no longer provide a solution, and people will start to discuss the controversial theory of utilitarian ethical theory.

As in *Outbreak*, after finding that the Motaba antiserum cannot save Henry (a lab technician) and Henry’s girlfriend, Brigadier General Ford realizes that “The virus has changed enough so that the antibodies cannot recognize it” [11] (p. 90). Torn between utilitarian and deontological ethics, Ford suggests Major General McClintock try to find the host in order to produce antibodies to the mutated strain. Ford listens to the brief of a biowar defense expert and learns that the epidemic has spread. Though angry at the uncontrollable situation (“Goddamnit!” [11] (p. 93)), Ford gives an order to increase the spray, trying his best to stop the spread of the Motaba infection. Nonetheless, Ford thinks that there has “got to be a way to deal with it” [11] (p. 93). However, being utilitarian, McClintock insists that “People can be asymptomatic, for weeks. The tests are faulty. We have to assume everyone in this town is infected and will get this disease sooner or later” [11] (p. 93). Torn between utilitarian and deontological ethics, Ford finally surrenders and follows Major General McClintock’s command to execute an emergency containment plan. After a silence, Ford gives a command: “I want the most rapid-acting nerve gas. Something so quick they’ll never know what hit ’em” [11] (p. 93). Though with pain clearly visible on his face, Ford commands his fellow soldiers:

Gentlemen, I have in my hand the final authorization to proceed, signed by the president. I know that each of us has doubts about what we are about to do. It is only human to have doubts when you are commanded to take the lives of other human beings. Remember your wives, remember your children...they will all get the disease and die if we let fear govern our hearts. We are doing what is right, and what the nation requires of us.

[11] (p. 122)

Being a solider, though torn between utilitarian and deontological ethics, Ford must follow his boss’s command, i.e., Major General McClintock’s command, a command that comes from the U.S. president.

To stop the virus from spreading, Colonel Daniels requests a quarantine in Cedar Creek. However, even in setting up a quarantine, without finding the host to develop the antiserum, there is still no way to stop the infection. Without antiserum, even firebombing Cedar Creek does not guarantee that the Motaba infection may not break out again someday. That is, firebombing would cause more pain and less welfare, which disobeys the principle of utilitarian ethics. As Daniels says, “If the monkey’s antibody is effective, you could use it as a template. Maybe you could save Europe” [11] (p. 125).

Colonel Daniels: If the antibodies work, we’d have a way to stop the epidemic by medical means.

Brigadier General Ford: We have no way to produce them in quantity.

Colonel Daniels: Dr. Iwabi.

Brigadier General Ford: There’s no time for that now. In the lead Apache, snipers lean out the window. Their telescopic sights lock on Gillespie and Salt.

Major General McClintock: Give me an order to fire, sir. We do not need the monkey.

Colonel Daniels: You do. As if you fail to contain the virus, you’re going to be racing to synthesize an antiserum. How long did it take you to do the last one? A year? Two years? Five? [11] (p. 125).

Hence, the race against two ticking time bombs becomes a race between utilitarian ethics and deontological ethics. Clearly, once again, Brigadier General Ford is torn between Colonel Daniels and General Major McClintock, between deontological ethics and utilitarian ethics. Ford falls into the ethical dilemma between whether Salt can successfully create the antiserum to save the Cedar Creek residents or whether he must resume Operation Clean Sweep, sacrificing few people in order to save the greatest number of humans.

Colonel Daniels: To the aircraft approaching Cedar Creek, this is Col Sam Daniels. I’m the doctor. You must not bomb this town.

Major General McClintock: Sandman. This is viper command, you are being spoofed by com chatter.

Colonel Daniels: Your commanders do not have the current data. We do. We have an antiserum being administered as we speak. Every infected person will have their dosage, so you must abort the mission. This is urgent! You cannot bomb this town! You must abort! I am talking to the pilots in the bomber. I know what you are about to do is not easy. However, I have told you the truth.

Major General McClintock: You will release that weapon on time and on target!

Colonel Daniels: Okay guys no more words. We are not moving from your path [9] (pp. 113:50–117:52).

In contrast, Daniels risks being sentenced or dismissed by the military, attempting to find the host of the virus to produce a vaccine for the epidemic. In order to protect the innocent, Daniels even flies a helicopter, intending to collide with the plane that is ordered to bomb Cedar Creek. Daniels is using his life to prevent innocent people from being harmed, underlining the principle of humanity values [19]. Fortunately, Betsy is captured in time and Ford delays the bombing to give Salt time to mix Betsy’s antibodies with the E-1101 to create the antiserum.

As Thomasma [4] notes, utilitarian theory has been criticized in that it cannot correctly predict the outcome of an action. Moreover, the action may not always turn out to have a desirable consequence, sometimes turning out to be evil instead. The firebombing of the residents of African jungle ignores individual rights and innocent people, but it may put an end to the Motaba infection; that is, the bombing may bring the least harm and the greatest happiness to the most people. However, the bombing cannot stop the infection but instead gives the virus a chance to mutate to become airborne. There is a difficulty in calculating the maximum utility for a given action, not knowing whether the action can lead to an increase or decrease in happiness or pain for the majority of human beings [25]. For instance, in *Outbreak*, initially, McClintock and Ford believe, as utilitarians, that that the bombing action in the African jungle guarantees the maximum benefit to human beings; however, the seemingly utilitarian action turns out to be an uncontrollable catastrophe. Therefore, it is not easy to justify the moral correctness of the action.

Facing an unknown virus, which appears in the middle of an African jungle during battle, a government would normally give medical care professionals time to make vaccines and stop the virus from spreading. However, a utilitarian may decide to achieve the fastest and biggest benefits: to use the bomb to wipe out the epidemic. McClintock and Ford definitely know that the only way to bring the greatest benefit to human beings is to find the host.

Colonel Daniels: You knew about Motaba all along. E-1101 was the antiserum. You could have stopped the outbreak before it mutated. You must tell me what the host is.

Brigadier General Ford: We have not found the host. We had to synthesize the antiserum.

Colonel Daniels: We could have stopped it then, but we do not because we must protect the perfect biological weapon. However, then the virus mutates and we cannot stop it now and we could have then.

Brigadier General Ford: The decision was made in the interest of national security. It was a terrible mistake to withhold E-1101, but we are beyond that now. We have done all we can as doctors. We must go on as soldiers.

Colonel Daniels: You are going to wipe out the town. You will eradicate that mutation and then your weapons intact [9] (pp. 78:43–80:43).

In *Outbreak*, in order to cover their biowarfare conspiracy, those deciding to firebomb the African jungle and Cedar Creek seem to hold the utilitarian ethics to seek the maximum welfare/happiness for the greatest number of humans. The government orders McClintock to bomb the village to stop the outbreak of the Motaba virus. However, the truth is that the army has already developed vaccines and wants to use the virus as a bioweapon in the future to maximize benefits for the government rather than for the greatest number of human beings.

Nowadays, it is impossible for medical professionals to fully adhere to certain medical ethics. As in the film *Outbreak*, the government is still the main force for making decisions during the virus pandemic; therefore, if, in real life, any incidents similar to the scenarios in *Outbreak* occur, many innocent people may be sacrificed because of a decision made based on utilitarian principles, i.e., to maximize the benefit of majority while sacrificing the innocent. In terms of the real-world scenario of coronavirus disease 2019 (COVID-19), the disease has broken out into a global pandemic, causing a crucial challenge to the public health and a heavy burden on healthcare systems [26]. Medical professionals are morally obliged to act for the good of all patients; they have managed to treat each individual patient fairly and indiscriminately. However, while facing unexpectedly influx of patients, with scarce medical resources, they have to follow governments’ coronavirus guidelines, policies, and action plans, such as the allocation of scarce ventilators, ICU beds, or other medical resources, in order to reach the optimal distribution of medical resources and to minimize mortality from COVID-19 [27]. These medical professionals’ ethical principles of protecting each individual from being hurt will inevitably collide with the realities of limited medical resources and governments’ COVID-19 action plans. Hence, in order to save the greatest number of humans, these medical professionals are caught in a dilemma between utilitarian ethics and deontological ethics.

However, what if governments deliberately hide or downplay the outbreak of COVID-19 or deliberately hide the spread of COVID-19 for some political or economic purposes? As shown by the government in *Outbreak*, which deliberately keeps the virus a secret to cover their biowarfare conspiracy, governments weighing economic development against the life of human beings would delay action plans to stop the spread of virus; hence, greater numbers of the population would be infected or sacrificed by the pandemic than would have otherwise been necessary. Moreover, the delay would also put medical professionals at higher risk of being infected.

Utilitarian ethics highlight the consequences of actions [12,13], trying to justify the actions that may bring the greatest amount of happiness for the greatest number of human beings. However, while attempting to reach the greatest happiness, an action may not respect each individual’s decision or, moreover, may sacrifice some innocent people. In other words, in order to attain the greatest happiness for the most people, the end is sufficient enough to justify the means; hence, any conventional moral thinking can be ignored [12,13,25]. However, deontological ethics respect individuals’ human rights and is not interested in results but in moral actions, which may sometimes lead to a reduction in the happiness of human beings [1]. Although some medical professionals are prone to utilitarianism, even if it seems unethical and morally wrong, there are still numerous doctors and medical care professionals worldwide acting correctly in relation to patients, as with Colonel Daniels and his team in *Outbreak*.

We can hypothesize that, one day, there will be a deadly virus, such as the Motaba virus in the film *Outbreak* or the present scenario of COVID-19, and there is a chance that this virus will spread all over the world. If there is no time or money to develop an antiserum, should we comply with deontological ethics or utilitarianism, choosing the fastest and most efficient way? To reach more just solutions, it is essential for medical professionals to reflect on how to make wise decisions by balancing deontological ethics and utilitarian ethics. However, the decision-making process is complicated because any solution must consider not only medical ethics but also political, environmental, and military issues.

## 9. Conclusions

As both utilitarian and deontological ethics hold their own perspectives in medical ethics, we cannot expect utilitarianism not to be involved in medical ethics. However, we can expect that, through education and policies, humans can clearly understand utilitarian ethics and hence strive for the spirit of utilitarianism. In order to reach a medical/healthcare decision, those involved should be inclined toward empathy and contemplate things from different ethical perspectives. In that way medical professionals can reach a balance, not a compromise, to deal with ethical and moral dilemmas and create greater beneficence and justice for patients and humans.

By analyzing the scenarios in *Outbreak*, readers and medical professionals may conceive of a possible real-life viral infection disaster, especially in the present coronavirus outbreak, and might empathize with the ethical dilemmas of making difficult medical and clinical decisions. While taking the film *Outbreak* as example, this study discusses these two controversial ethical issues, deontological ethics and utilitarian ethics, in relation to the ethical handling of *Outbreak*’s scenarios and the present scenario of COVID-19 to stimulate readers’ and medical professionals’ reflection upon medicine and ethics. When medical professionals manage to address the conflicts between deontological and utilitarian ethics, they may reach a good balance between these ethics and create a more harmonious and justified medical practice for patients and others involved.

## Data Availability

Not applicable.
